# The continual threat of influenza virus infections at the human–animal interface: What is new from a one health perspective?

**DOI:** 10.1093/emph/eoy013

**Published:** 2018-09-07

**Authors:** Emily S Bailey, Jessica Y Choi, Jane K Fieldhouse, Laura K Borkenhagen, Juliana Zemke, Dingmei Zhang, Gregory C Gray

**Affiliations:** 1 Duke Global Health Institute, Duke University, Durham, NC, USA; 2 Division of Infectious Diseases, Duke University School of Medicine, Durham, NC, USA; 3 School of Public Health, Sun Yat-sen University, Guangzhou, Guangdong Province, China; 4 Global Health Research Center, Duke-Kunshan University, Kunshan, China; 5 Emerging Infectious Diseases Program, Duke-NUS Medical School, Singapore

**Keywords:** influenza, One Health, emerging viruses, zoonoses

## Abstract

This year, in 2018, we mark 100 years since the 1918 influenza pandemic. In the last 100 years, we have expanded our knowledge of public health and increased our ability to detect and prevent influenza; however, we still face challenges resulting from these continually evolving viruses. Today, it is clear that influenza viruses have multiple animal reservoirs (domestic and wild), making infection prevention in humans especially difficult to achieve. With this report, we summarize new knowledge regarding influenza A, B, C and D viruses and their control. We also introduce how a multi-disciplinary One Health approach is necessary to mitigate these threats.

## INTRODUCTION

In 2018, we mark the centennial of the 1918 influenza pandemic; an event that caused an estimated 20–50 million deaths worldwide [1]. The severity of the 1918 pandemic was a result of several important factors including a lack of pre-existing immunity to this newly emerged virus, as well as overcrowding, poor sanitation and the lack of antibiotics. However, despite the production of annually designed vaccines and the many improvements in public health surveillance and infrastructure, each year in the USA alone, seasonal influenza A and B viruses continue to evolve and take the lives of 3000–48 000 people [2]. It is now also clear that other related viruses (influenza C and D viruses) may also cause at least subclinical infections in humans. In short, the ecology of influenza viruses is recognized today as one of the most complex and difficult to mitigate public health problems.

A key component to this complexity is the observation that influenza A, B, C and D viruses may have numerous animal species reservoirs (domestic and wild), making their infection prevention in humans especially difficult to achieve. It also seems clear in at least recent years that the most important influenza A virus threats to humans are often amplified in domestic animals. Modern agricultural practices, such as the worldwide shift towards raising pork and chickens in confined animal feeding operations (CAFOs) may be amplifying these public health threats [3]. It is also very relevant that human-reservoired influenza viruses are being introduced into swine CAFOs and sometimes causing large and important clinical disease outbreaks among the pigs [4, 5]. Some might argue that the solution is to raise pigs in less animal-dense farms but this position is simply not realistic. It is through modern agricultural techniques, that include CAFOs, that most nations are seeking to produce increase meat production to the increasing demands of rapidly growing human populations.

Hence, we need to find new ways to engage professionals in human health, animal health, environmental health and agricultural businesses to work together to study the ecology of influenza viruses, and to jointly develop and test interventions to reduce their risk to humans and animals. This is the premise behind the One Health approach. Although the interconnections of humans and animals have long been recognized [6], One Health was first officially recognized by the American Veterinary Medical Association in 2007. One Health calls for deliberate and focused collaborations between experts in the animal, human and environmental health fields. A holistic and versatile One Health approach is now recognized as imperative in fostering the effective communication and actions needed to respond to influenza virus threats. Over the last 100 years, this approach has evolved from isolated disciplines working together only in times of pandemic to the continued push today for collaboration from public health workers, medical professionals, veterinarians and many others. This shift has changed the way influenza, and pandemic influenza in particular, are approached with an increase in communication, partnership and outreach when tackling these issues.

**Figure 1.**
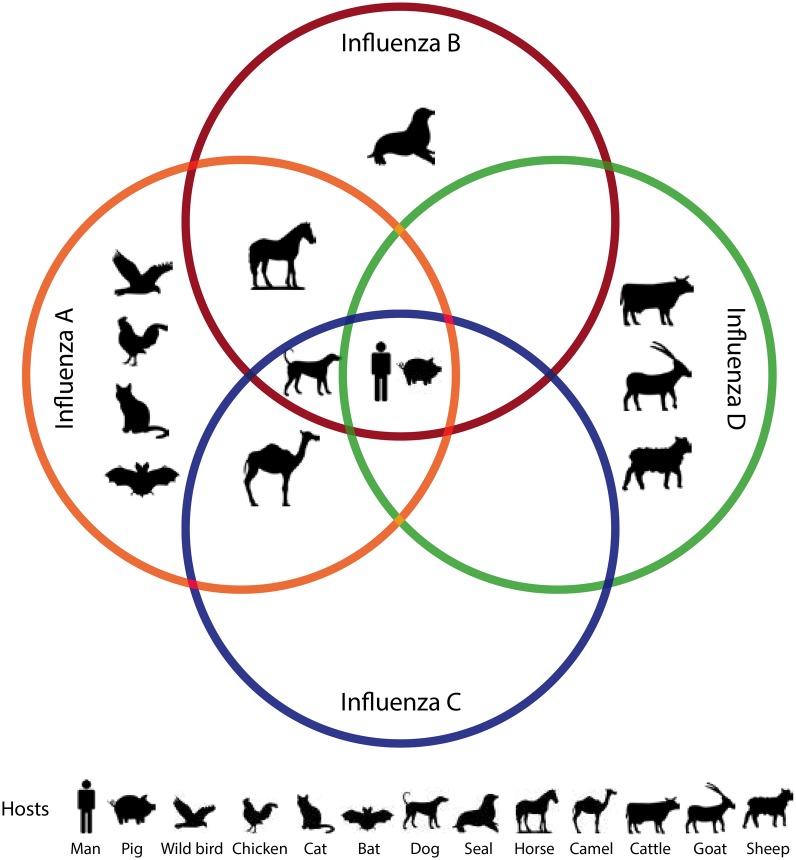
Graphical summary of the reports of human and animal infections with the various influenza viruses (Genera influenza virus A, B, C, & D). It is interesting to note that humans and pigs are thought to be susceptible to all four influenza genera. Among the animals with documented influenza infections, many are domestic animals. In particular, poultry and pigs serve as important amplifying reservoirs for influenza A virus infections in man

Evolutionary medicine is a discipline that uses evolutionary theory to understand health and disease [7]. The central focus of this framework is that selection acts on fitness and that evolution and adaption alone do not cause disease. One Health and evolutionary medicine are interconnected in that both require an interdisciplinary approach to complex problem solving. Often both disciplines work at the intersection of changing environments, animal and human habitats. In particular, with the case of influenza, the continued evolution and adaptation to new and diverse host species, as shown in Figure 1, presents a unique problem that requires the use of both disciplines.

With this report, we will outline newly described influenza virus types and subtypes and novel developments in influenza virus detection and control. We also introduce how an interdisciplinary One Health approach seems the best way forward to mitigating these threats.

## INFLUENZA A VIRUS

Influenza A virus (IAV) [8] is highly infectious and can be transmitted to humans via direct contact with secretions, aerosolized respiratory droplets and indirect contact with fomites. Known for its high morbidity and mortality rates, IAV is the cause of both seasonal and historic influenza pandemics, including the severe 1918 H1N1 pandemic, the 1957 H2N2 ‘Asian Flu’ pandemic, the 1968 H3N2 pandemic, and most recently the 2009 novel H1N1 virus pandemic, the latter of which likely emerged from domestic pigs resulted in between 151 700 and 575 400 human deaths worldwide [9].

More recently, influenza activity during the 2017–18 influenza season has been the highest yet since the 2009 pandemic, with a cumulative incidence rate of 59.9 influenza-hospitalizations per 100 000 people in the USA [10]. IAVs constituted 84% of the more than 51 000 specimens tested in public health laboratories across the USA and Puerto Rico between October of 2017 and February of 2018 [10]. Of the IAV subtypes, 89.9% were IAV H3N2 viruses and 10.1% were IAV H1N1 viruses. More than 71% of the 63 influenza-associated pediatric deaths this 2017–18 influenza season were associated with IAV infections, a slightly higher proportion than the overall proportion (65%) of pediatric deaths attributable to IAV infection between 2010 and 2016 in the USA [10].

Growing numbers of avian-origin IAVs, which include 16 of the 18 hemagglutinin glycoprotein subtypes and 9 of the 11 neuraminidase (NA) subtypes, are increasingly found to infect humans. Due to increases in the number of human infections with avian-origin H7N9 IAV in China in 2017, a study of 40 case-patient clusters across 5 recent epidemics from 2013 to 2017 sought to determine if human-to-human transmission of H7N9 has increased in the past 5 years. While the study found no change in the human-to-human transmission of H7N9 over time, among the 40 clusters, 35% were classified as probable and 65% as possible human-to-human transmission [11]. Sustained human-to-human transmission, however, has not been seen between epidemics, suggesting that non-human animals are important for maintenance of this virus. Humans infected with H7N9 typically present with severe symptoms and the mortality rate is ∼40%.

Environments that facilitate IAV include birds [12], pigs [13], horses [14], dogs [15] and most recently bats [16]. In particular, aquatic birds are considered the primordial reservoir of all influenza viruses for avian and mammalian species. Evidence continues to support the assertion that environments that facilitate the interaction of human and avian species, such as the live poultry markets, often increase transmission risk to humans [12]. During the 2013 and 2014 IAV (H7N9) outbreak in China, for example, the majority of patients with laboratory-confirmed cases of IAV (H7N9) reported recent exposure to live poultry markets [17]. A type of avian IAV (H7N4) was detected in a human patient, with a reported history of contact with live poultry, for the first time in February of 2018, prompting authorities to be on alert for the pandemic potential of the virus [18]. Additionally, multiple studies have provided evidence of bidirectional transmission of IAV at the interface of human and pig populations, especially at agricultural fairs [13, 19]. Asymptomatic pig entrants in these public exhibit spaces are suspected viable facilitators for IAVs to jump between species [13]. Compared with other influenza types, the zoonotic transmission of IAV has been frequently documented and findings on the trans-species paths of infection indicate the expanding potential for new strains of IAV to emerge at the human and animal interface. There will likely be a continued need for effective control and prevention of emerging zoonotic IAV. As such, surveillance efforts for IAV would benefit from a One Health approach that would employ surveillance at the human–animal interface where novel subtypes are most likely to threaten the human population.

A select few antivirals have been approved and demonstrated effective to reduce symptoms of IAV and concurrently reduce transmission. These are summarized in Table 1. Oseltamivir, peramivir and zanamivir are three NA inhibitor antiviral interventions. Two M2 ion channel inhibitors (amantadine and rimatadine) have also been approved.

**Table 1.** Characteristics of influenza viruses

Virus type	Year of virus discovery	Number of gene segments	Available antiviral therapy	Seasonal vaccine routinely available
Influenza A	1931	8	Oseltamivir, peramivir, zanamivir, amantadine, rimatadine	Yes
Influenza B	1940	8	Oseltamivir, zanamivir	Yes
Influenza C	1974	7	No effective antiviral treatment available	No
Influenza D	2011	7	No antiviral treatment available	No

Evolutionary perspectives are important in considering treatment options. For example, most IAV strains have developed resistance to the M2 ion channel blockers; thus, amantadine drugs are not currently being recommended for preventing or treating influenza [20]. While NA inhibitor medications are the current recommended interventions, concerns are growing over the emergence of oseltamivir-resistant strains. Since October 2017, the CDC found 1.1% of 376 H1N1 IAV strains are resistant to both oseltamivir and peramivir; however, all 903 H3N2 viruses tested were susceptible to both oseltamivir and zanamivir [10]. New monoclonal therapies are under development which may increase therapeutic options for patients with severe IAV disease [21].

For healthy adults, annual trivalent or quadrivalent influenza vaccines have been the major prophylactic prevention mechanism for several decades. In order to inform the selection of the viral composition of season vaccines, robust active surveillance and epidemiological and evolutionary modeling is required to predict the upcoming seasonal strains that will circulate in the Northern and Southern hemispheres. Mismatches can occur, due in part to frequent minor changes in virus glycoproteins occurs between the time when the strains are selected and the time the vaccine is produced, reducing the effectiveness of the vaccine as was the case during this 2017–18 influenza season. Current vaccine effectiveness (VE) estimates of the 2017–18 vaccine are estimated at 36% overall with 25% VE specifically against H3N2-related illness and 67% VE against H1N1 viruses [22]. When considering vaccine options and effectiveness, there are several important scientific and policy considerations related to availability, access and prevention of future disease. From a One Health perspective, other virus hosts and reservoirs of IAV should be considered in the development of future vaccines as well as the surveillance of resistance in the environment.

## INFLUENZA B VIRUS

In the spring of 1940, influenza B virus (IBV) was first identified from a child during an acute respiratory epidemic in the United States [23]. Since then IBV strains have been recognized to cause considerable seasonal morbidity and mortality with B/Victoria/2/87-like (Victoria lineage) and B/Yamagata/16/88-like (Yamagata lineage) strains being the most prevalent [24]. During influenza seasons, IBV, along with IAV (H1N1) or H3N2 co-circulate and become the most prevalent strains every 2–14 years [25]. Compared with IAV, IBV has less antigenic variation and fewer subtypes [26]. Despite being considered less of a public health threat than IAV, IBV has been reported with prevalence of up to 82.4% among individuals reporting influenza-like illness (ILI) [27] and symptoms such as encephalitis, myositis, even death have been previously reported [28].

IBV is mainly associated with human infection [29]; however, other animal reservoirs have been proposed, suggesting that a One Health perspective is also important for this influenza virus. Pigs are susceptible to IBV and may serve as a natural reservoir [30]. Additionally, antibodies against IBV have been isolated from horses and pigs in Japan [31] and from dogs in Taiwan [32]. In 1999, influenza B was isolated from a harbor seal with a respiratory disease that was associated with a large seal die-off in the Netherlands. Phylogenetic analyses of viruses obtained from seal serum indicated that the IBV was of human origin [29]. Thus far, no novel seal-reservoired IBV strains have been detected and no evidence of IBV seal to human transmission has been reported.

At present, there are two approved antiviral drugs (NA inhibitors oseltamivir and zanamivir) for IBV infection [33]. Oseltamivir is the most widely administered for the prophylaxis and treatment of IBV infection in patients older than 1 year [34]. Zanamivir is administered to patients older than 7 years by inhalation and functions directly in the respiratory tract [34]. Several studies show that oseltamivir is less effective in treating IBV compared with the treatment of IAV [35], zanamivir was equally effective for IAV and IBV infections and more effective than oseltamivir for the treatment of IBV [35]. However, evolutionarily new NA inhibitor (NAI)-resistant IBV viruses pose a public health concern as they are not susceptible to oseltamivir or zanamivir [36]. IBV variants, including R152K, D198N, G109E and G402S, R152K have been identified as NAI-resistant [36]. In addition to antiviral therapies, currently a quadrivalent influenza vaccine, including two influenza A subtypes (H1N1 and H3N2) and two influenza B lineages (Victoria and Yamagata) are available [37].

As IBVs continue to pose a significant risk to the public, particularly for children and the immunocompromised, better therapies for IBV are greatly needed. From an evolutionary perspective it is important to choose vaccines and therapies that are relevant to the new and evolving viruses and further research should be targeted to limit further development of resistance or virulence in IBV variants. Similarly from a One Health perspective, it is important to choose vaccines that incorporate information from new and novel strains that emerge from host species.

## INFLUENZA C VIRUS

First identified in humans in 1974, the most common reservoir for influenza C virus (ICV) is humans, with up to 80% of individuals acquiring antibodies to ICV by the age of 7–10 years [38]. Although ICV infections are typically reported at extremely low frequency, they are reported consistently. A study of Eastern Indian patients with acute respiratory illness reported that 0.18% were ICV-positive from January 2011 to December 2012 [39]. In Scotland, ICV was present in 0.2% of the 3300 human respiratory samples among patients <2 years or >45 years old during the summer and winter between 2006 and 2008 [40]. ICV outbreaks were also reported in Singapore, Japan and France between 2004 and 2007 [41]. Additionally, ICV epidemics have been reported in Australia approximately every 2 years during the years of 2010, 2012 and 2014 [42]. One proposed reason for this pattern involves evolutionary change, driven by a high frequency of reassortment in ICV.

ICV has been known to naturally infect domestic pigs [43] and feral dogs [44]. When feral dogs were nasally infected with human ICV, they developed clinical symptoms while shedding the virus for more than 10 days, suggesting that dogs may serve as natural reservoirs for human ICV [44]. In addition, in a 2017 ICV antibody study, dromedary camels in Kenya were suspected to serve as a newly recognized host for ICV as they were found to harbor ICV antibodies [45]. This recent discovery suggests that ICV, such as IAV and IBV, may have a wider host range than previously thought. Thus, a One Health approach to surveillance among animals and among environments is needed for ICV, especially where extended interactions between animals and humans may favor spillover of the virus.

As the symptoms associated with ICV are less severe in comparison with other forms of influenza and respiratory infection [46], less attention has been drawn to developing antivirals and vaccines against ICV. Hence there are no effective antiviral treatments or vaccines available for ICV [42]; however, in the development of future therapies for ICV, it will be important to take a One Health approach to evaluate the animal reservoirs for this virus and their potential impact on the spread of the virus in humans.

## INFLUENZA D VIRUS

In 2011, a viral isolate with ∼50% amino acid homology to human ICV was collected from a pig exhibiting ILI in Oklahoma [47]. Although initially believed to be a subtype of ICV, this virus has now been recognized as a new genus in the *Orthomyxoviridae* family: influenza D virus (IDV). Antibodies of IDV have been detected in pigs, cattle, goats and sheep. In animals, the highest prevalence of IDV has been detected in cattle with symptoms of bovine respiratory disease, especially in calves (6–8 months) due to their underdeveloped immune system [48]. Serological evidence indicates IDV has been present in US cattle populations since as early as 2004 [43]. A cross-sectional serological study of cattle-exposed adults in Florida found a high prevalence (97%) of neutralizing antibodies compared to non-exposed controls (18%) suggesting occupational exposure risk [49]. Similarly, a study conducted on cattle in Mississippi showed 94% seroprevalence in neonatal beef cattle in addition to IDV transmission in comingled cattle herds [50]. During a swine respiratory disease outbreak in Northern Italy in 2015, the IDV genome was detected and isolated in both pigs and cattle herds [51]. The IDV genome isolated from the pigs was closely related to the viral genome isolated in the United States in 2011. Additionally, the archived serum samples from 2011 had lower IDV antibody titers when compared with the serum samples collected in 2015, suggesting that the incidence of IDV infections in pigs may have increased over time, and therefore, IDV may pose a public health threat to the community.

Relatively little is known about the potential zoonotic transmission of IDV to humans [47], and to date the signs and symptoms of acute IDV in humans have not been described. With the increased potential of IDV transmission in and between animal reservoirs, and the high presence of neutralizing antibodies in cattle exposed workers, there is a need to determine if IDV is zoonotic through One Health oriented surveillance. Currently there is no recommended therapy or vaccine available for IDV.

## CONCLUSION AND FUTURE DIRECTIONS

Since the 1918 influenza pandemic, there have been many changes to public health infrastructure as well as new developments in vaccine technologies. Despite this, influenza viruses exhibit remarkable evolutionary change and adaptation to new animal hosts. The One Health approach has been proposed as a way to work across disciplines to incorporate human, animal and environmental health in order to solve complex problems, such as infectious disease outbreaks. Novel One Health strategies for future surveillance may include bioaerosol surveillance at the human–animal interface. These alternative strategies are advantageous due to low cost, less invasive sampling methods that are acceptable to industry [52–56].

Today, novel research on influenza viruses is conducted not only by medical doctors and vaccines scientists, but also veterinarians and the agricultural industry seeking to reduce influenza virus morbidity in animal hosts. Additionally, during the 2009 H1N1 pandemic the global community was informed and a vaccine was prepared in record response time as a result of international, multidisciplinary collaboration.

In the future, it will be increasingly important for multiple disciplines to collaborate in studying influenza viruses in an effort to mitigate influenza virus outbreaks in both humans and animals. As these viruses continue to evolve, particularly in relation to virulence, resistance and ecology, there is a need for rigorous collaboration using the One Health approach to prevent not only future outbreaks but also to track the spread of infectious disease.

## Funding

This study was supported in part by NIH/NIAID grant R01AI108993-01A1 (Gray PI). 

**Conflict of interest**: None declared.
